# Biogenic Zinc Oxide Nanoparticles as a Promising Antibacterial Agent: Synthesis and Characterization

**DOI:** 10.3390/ijms25179500

**Published:** 2024-08-31

**Authors:** Kunle Okaiyeto, Maria Rosa Gigliobianco, Piera Di Martino

**Affiliations:** Department of Pharmacy, University of “G. d’Annunzio” of Chieti and Pescara, Via dei Vestini, 1, 66100 Chieti, Italy; okaiyeto.kunle@unich.it (K.O.); piera.dimartino@unich.it (P.D.M.)

**Keywords:** green synthesis, zinc oxide nanoparticles, phytochemical compounds, antibacterial activity, biogenic nanoparticles

## Abstract

Nanotechnology has gained popularity in recent years due to its wide-ranging applications within the scientific community. The three main methods for synthesizing nanoparticles are physical, chemical, and biological. However, the adverse effects associated with physical and chemical methods have led to a growing interest in biological methods. Interestingly, green synthesis using plants has gained prominence in developing new treatments for bacterial infections. Zinc oxide nanoparticles (ZnO NPs) produced using environmentally friendly methods are more biocompatible and have potential applications as antibacterial agents in the biomedical field. As a result, this review discusses the green synthesis of ZnO NPs, factors influencing optimal synthesis, characterization techniques, and the antibacterial activity of some plant-mediated ZnO NPs. It also provides a comprehensive and analytical exploration of ZnO NP biosynthesis, the role of phytochemical compounds as reducing and stabilizing agents, the mechanism of action of their antibacterial properties and further highlights the challenges and prospects in this innovative research area.

## 1. Introduction

Nanotechnology has garnered significant attention due to its cutting-edge capabilities in producing materials on a nanoscale (1–100 nm) [1,2,3]. Over the last several decades, several industries, including food, agriculture, pharmaceuticals, medicine, and others, have exploited metallic oxide nanoparticles [4]. Zinc oxide (ZnO), among other components, has been identified by the Food and Drug Administration (FDA) as a safe, or Generally Recognized As Safe “GRAS”, chemical and is an important semiconductor [5]. Zinc oxide (ZnO) is an inorganic compound utilized in sunscreens, paints, pharmaceuticals, and ceramics, among other applications [6]. Zinc oxide is an excellent economic and commercial choice due to its unique physicochemical features, thus making it suitable for various applications [7]. Remarkably, ZnO NPs have lately gained prominence in the scientific community because of their several potential applications, especially in biology, the environment, and electronics [8]. ZnO NPs are widely investigated for use in a multitude of applications among metal NPs owing to their optical properties, low cost, safety, biocompatibility, minimal cytotoxicity, ease of manufacture, semi-conductivity, piezoelectric, spintronic, photonic abilities, vulcanization stimulator, and antibacterial activity [9,10]. Considering their strong excitonic binding energy (60 meV) and broad and straight band gap semiconductor (3.37 eV) [3,11], ZnO NPs can function well in optical systems at or above room temperature [12]. Owing to their distinctive ability to disperse UV rays, they are often utilized in sunscreen [13]. In a recent study by Jha et al. [14], it was revealed that ZnO NPs demonstrate exceptional biocompatibility, minimal toxicity, and cost-effectiveness, further solidifying their extensive potential for biological applications.

The preparation of nanoparticles is an important topic that has recently gained popularity. There are three methods for synthesizing ZnO NPs: physical, chemical, and biological [15]. However, the high energy consumption and pressure required to generate NPs via a physical approach make the production costs too expensive, and this method involves the use of sophisticated equipment and well-trained personnel to operate; therefore, its usage for production is discouraged [16]. Furthermore, chemical methods are conventionally used because the cost of their synthesis is less expensive and involves the use of light equipment compared to physical methods. Nevertheless, the chemicals used as reducing agents and stabilizing or capping agents in chemical methods are toxic and not eco-friendly; as a result, this adverse effect necessitates searching for safer alternatives that are less toxic, simple, sustainable, economically viable, and environmentally benign [17].

This review focuses on the biological synthesis of ZnO NPs and the factors that influence successful synthesis and characterization. In addition, the antibacterial properties of various plant-mediated ZnO NPs were summarized, and the mechanism of antibacterial action was articulated. The constraints and opportunities in providing significant insights into this emerging research topic were also highlighted.

## 2. Green Synthesis of ZnO NPs

**Figure 1:**
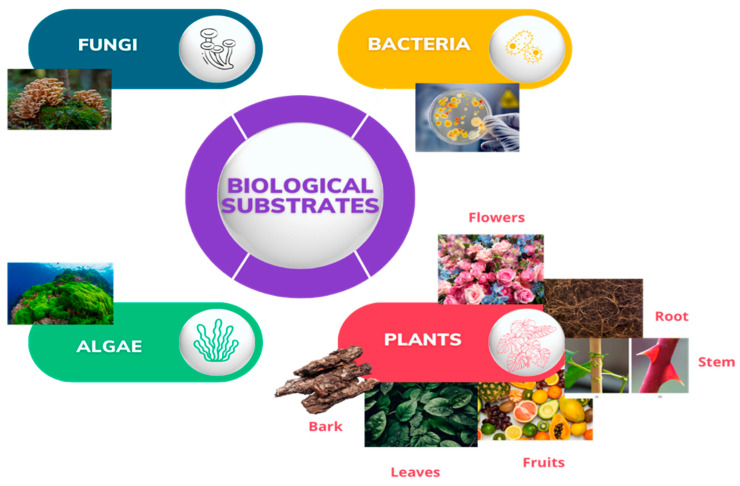
Biological substrates used for green synthesis of ZnO NPs.

To achieve sustainable development goals and reduce waste, there is an urgent demand for green nanoparticle production technologies [18]. One of the primary purposes of using green sources to produce nanomaterials is to harness natural resources to solve environmental challenges [1]. Biological synthesis stands as a superior bionanotechnology tool, replacing chemical and physical processes. It offers cost-effectiveness, environmental friendliness, speed, simplicity, and economic viability [10,19]. In this method, non-toxic chemicals that are environmentally acceptable are employed [17,20]. According to the report by Rahman et al. [21], advances in nanotechnology help decrease pharmaceuticals’ adverse effects on human health and improve the clinical efficacy of medicine. Leveraging nanobiotechnology alongside therapeutic research has significantly boosted the potency of therapeutic molecules [22]. In green synthesis, substrates used include different plant parts (leaves, fruits, flowers, stem, rhizome, and aerial), bacteria, fungi, and marine algae (Figure 1). By employing plants, bacteria, fungi, and algae as substrates, an environmentally friendly synthesis process can be achieved that effectively lowers the toxicity of both the final product and the manufacturing process [14]. The exploration of microorganisms for green synthesis of nanoparticles has been established to be associated with some limitations, including contamination from culturing and cost implications [23].

On the contrary, because they promote the large-scale production of stable nanoparticles in a variety of sizes and forms, plants offer the simplest and ecologically benign method for synthesizing nanoparticles [24,25,26]. Plant extracts contain phytochemical compounds that can serve as both stabilizing and reducing agents. The utilization of plants for green synthesis has drawn a lot of biotechnological attention [27,28]. Different secondary chemical components, including carbohydrates, alkaloids, amino acids, glycosides, phenolic compounds, oils, lipids, and saponins, may be present in the plant extract. The green synthesis method produces nanoparticles (NPs) that are functionalized with various phytochemicals. This enhances the biocompatibility and bioactivity of the NPs, making them effective for many biomedical applications [29]. It has been reported that using biomolecules as reducing agents can cause particle aggregation due to the electrostatic interaction between metal ions and biomolecules [5]. These biomolecules help control the size and shape of the particles, functionalize the surface of the NPs, and enhance their biocompatibility and bioactivity [30].

Green synthesis offers a powerful approach to producing nanoparticles (NPs) that are functionalized with diverse phytochemicals. This method enhances the biocompatibility and bioactivity of NPs, making them ideal for a wide range of biomedical applications [29]. It has come to light that utilizing biomolecules as reducing agents can lead to particle aggregation because of electrostatic interactions between metal ions and biomolecules [5]. These biomolecules play a crucial role in regulating particle size and shape, modifying the surface of the NPs, and amplifying their biocompatibility and bioactivity [30].

The green synthesis method, an environmentally friendly approach, involves the use of plant-derived phytochemicals to functionalize nanoparticles (NPs). This process results in NPs with enhanced biocompatibility and bioactivity, making them highly suitable for a myriad of biomedical applications [29]. It has been observed that the utilization of biomolecules as reducing agents can lead to particle aggregation due to electrostatic interactions between metal ions and biomolecules [5]. These biomolecules play a pivotal role in not only controlling the size and shape of nanoparticles but also in modifying the surface of NPs, thereby enhancing their biocompatibility and bioactivity [30]. Research has primarily been conducted in a controlled laboratory environment, where a range of experiments has been carried out to explore the potential applications of plant-based extracts in the synthesis of zinc oxide nanoparticles. This research has generated substantial interest in commercial sectors due to the reduced need for heavy equipment and the considerable advancements in our understanding of the composition of biological extracts and their interactions with metal ions.

**Figure 2:**
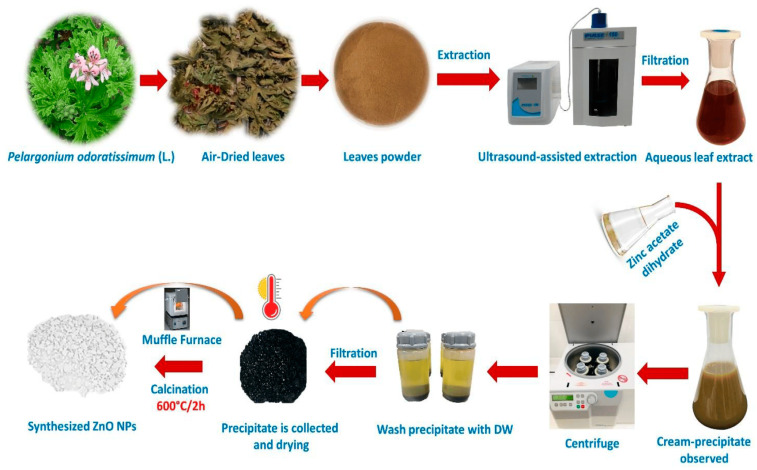
A graphical representation of the green synthesis of ZnO NPs using an aqueous plant extract. Figure reuse from [27].

A plethora of studies have demonstrated the successful synthesis of biogenic ZnO NPs from various plant extracts, as evidenced by previous studies conducted by other researchers [31,32]. Moreover, the proven antibacterial effects of ZnO NPs against both gram-positive and gram-negative bacteria have generated substantial interest in their potential uses [5,33,34,35]. For example, a green synthesis of ZnO NPs is represented in Figure 2.

### 2.1. Optimization of Green Synthesis Conditions

Several important factors influence the synthesis of ZnO NPs through plant-mediated methods. These factors encompass the concentration of biomass, or the volume of plant extract utilized in the synthesis, the specific pH level of the reaction mixture, the duration required for the completion of the nanoparticle formation process, the temperature during the reaction, the subsequent calcination temperature, and the precise concentration of salt present. Each of these factors plays a significant role in dictating the outcome of ZnO NP synthesis, and their careful consideration is essential for achieving the desired results. Optimizing these growth components to achieve the necessary NP size and shape for successful biological applications plays an important role [1]. The most widely acknowledged method for optimizing key factors responsible for biomolecule synthesis is response surface methodology (RSM). It is successfully used to optimize conditions in food, chemical, and biological processes [36]. Employing RSM has the benefits of saving raw materials, time, and space. It also produces a mathematical model (Box–Behnken design) that accurately describes the entire procedure, excluding the analysis of the impact of independent factors. Several other researchers have indicated that they have optimized several parameters in RSM-based nanoparticle production [37,38]. According to the report by Nithya et al. [39], physicochemical factors such as pH, temperature, and incubation duration are important for the microbial-mediated production of nanoparticles employing RSM. Typically, in green synthesis, a metal salt, such as zinc salt, is mixed with aqueous plant extracts, and the mixture is subjected to magnetic stirring for some time, and a colour change indicates the formation of nanoparticles [1]. Optimization of the following parameters is necessary to achieve the best yields: pH, time, temperature, extract concentration, and salt concentration [1].

#### 2.1.1. pH

**Figure 3:**
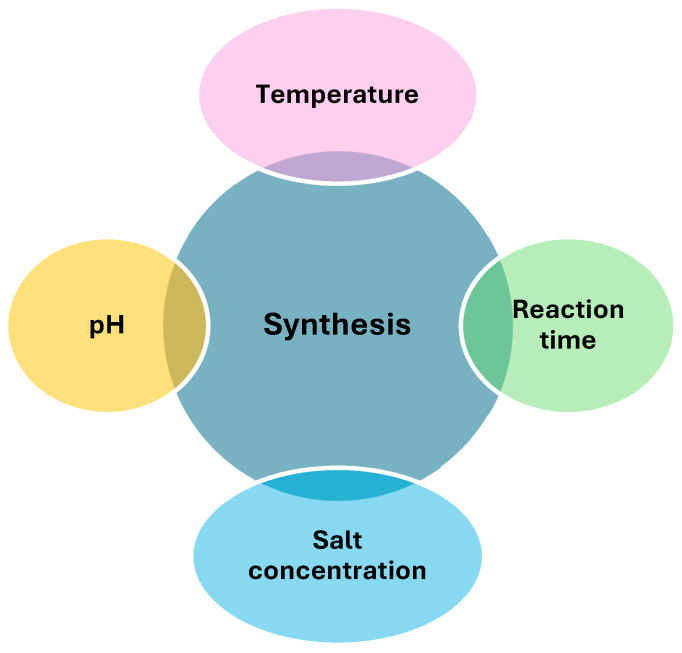
Factors influencing the biosynthesis of ZnO NPs.

One of the most significant effects of reaction pH is its ability to modulate the electrical charges of biomolecules. This modulation can have a profound influence on the biomolecules’ capacity to encapsulate and stabilize nanoparticles and subsequently contribute to the development and growth of the nanoparticles [40]. The development of nucleation centers is additionally controlled via temperature and pH. As the pH level rises, the number of nucleation centers increases simultaneously, which are the sites where the formation of metal nanoparticles begins. This increase is crucial as it promotes and enhances the formation of metal nanoparticles. Additionally, it is widely recognized that pH plays a major role in determining both the size and the structural shape of the nanoparticles [41]. Arif et al. [42] reported the synthesis of *Clinopodium vulgare* L.-mediated ZnO NPs at pH 9. Fernandes et al. [43] reported the synthesis of *Terminalia catappa* Fruit Pericarp-mediated ZnO NPs at pH 10. Notably, their research sheds light on the intricate process of nanoparticle synthesis and its correlation with pH levels. Another study investigated by Naiel et al. [44] synthesized ZnO NPs using *Limonium pruinosum* L. Chaz. extract at pH 8. Furthermore, existing literature by other researchers documents diverse instances of ZnO NP synthesis spanning pH levels from 8 to 12 [3,45,46]. This range underscores the significance of pH as a pivotal factor in the synthesis of ZnO NPs, warranting further exploration and elucidation in this area of study (Figure 3).

#### 2.1.2. Temperature

Temperature has a significant effect on the synthesis of nanoparticles (Figure 3). Several studies have highlighted that an increase in temperature increases the rate of synthesis of nanoparticles (reduces synthesis time) [47]. Higher temperatures can cause the breakdown of active phytochemicals found in the plant extract used for synthesis [48]. According to previous studies, the optimum temperature for zinc oxide nanoparticles ranges from 60 to 80 °C [49]. This optimum temperature results in maximum absorbance at the characteristic ʎmax of ZnO NPs. High temperatures (>100 °C) produce larger particles (60–100 nm). On the other hand, one possible explanation for the improved nanoparticle sizing at low temperatures may be a decrease in the growth and aggregation of the nanoparticles. Kahsay [50] states that the biosynthesis of ZnO NPs is not adversely affected by low temperatures and short synthesis periods. *Pistacia lentiscus*-mediated ZnO NPs were synthesized at 78 °C [51], while ZnO NPs using *Myristica fragrans* were conducted at 60 °C [52].

#### 2.1.3. Salt Concentration

The salt concentration used in ZnO NP synthesis influences their morphological features and size (Figure 3) [53,54]. The production of nanoparticles is also significantly influenced by the concentration of metal ions, which are usually obtained from metal salt solutions [55]. Variations in metal ion concentration can alter particle size, distribution, and composition by influencing the nucleation and growth processes. Increased metal ion concentrations have the potential to accelerate particle nucleation and growth, but they can also lead to unintended crystal formations or increased particle aggregation. The stability and optical characteristics of the nanoparticles can also be affected by variations in the concentration of metal ions. Controlling the properties of nanoparticles requires determining the optimum concentration of metal ions [55]. The higher the salt concentration (0.1–2 M) used, the bigger the size of the nanoparticles and vice versa. The size of the nanoparticles has a great influence on biological activity, and several researchers have highlighted in the literature that the smaller the nanoparticles, the higher the therapeutic potential because small-sized nanoparticles can transport faster across biological membranes as compared to the larger ones [56,57,58].

#### 2.1.4. Reaction Time

The reaction conditions greatly influence the physicochemical properties of nanoparticles through green synthesis (Figure 3) [59,60]. Therefore, the effects of synthesis conditions and associated factors on the reactivity, structure, and other features of the generated nanoparticles are investigated. The reaction time for plant-mediated synthesis of nanoparticles differs from one plant to another, the reason being the variations in the phytochemical compounds in different plants [61]. The types of phytochemical compounds in plants differ because of differences in climatic conditions, soil types, and other factors that play a significant role in the variation of these secondary metabolites. The level of stress to which these plants are exposed can influence the concentration of secondary metabolites they produce to protect them. It has been reported in the literature that when plants are exposed to certain stress, they secrete secondary metabolites for protection [62].

The use of plant extracts as reducing agents in green chemistry has been widely accepted by researchers in this field, and the reaction time for the phytochemical compounds in these plants to reduce metallic salts to nanoparticles depends on their type and concentrations. The compounds contained within the extract play a crucial role in influencing both the size and size distribution of metallic nanoparticles. The extract’s powerful reductant significantly accelerates the reaction rate, which in turn leads to the formation of smaller nanoparticles, as demonstrated in a study by Roy et al. [63]. Several researchers have reported in the literature that some plants have the potential to mediate the reduction of metallic salts to nanoparticles immediately, and others have documented different times for the completion of nanoparticle synthesis [16,64,65]. Combining different plants usually reduces the reaction time, as the synergistic effect of the phytochemical compounds in the plants aids in faster bioreduction of metallic salts to nanoparticles compared to single plants [25]. The longer the reaction time for the synthesis of nanoparticles, the larger the synthesized nanoparticles tend to come together and aggregate to form larger particles [55]. Plant extracts include phytochemicals that are primarily responsible for increasing dispersion and ultimately reducing agglomeration [66].

### 2.2. Purification of ZnO NPs

Purification of plant-mediated ZnO nanoparticles is a crucial step in green synthesis. After observing the complete reduction of zinc salts to zinc oxide nanoparticles, white precipitates usually form and settle at the bottom of the flask used for the synthesis. These precipitates are subsequently collected via centrifugation; for example, Mbenga et al. [67] reported collecting ZnO NPs synthesized using an extract of *Tulbaghia violacea* at 4300 rpm for 30 min, whereas Gamedze et al. [68] documented collecting ZnO NPs synthesized using an extract of *Mucuna pruriens* (utilis) at 5000 rpm for 30 min. The precipitate is then resuspended and thoroughly washed with distilled water to remove any associated impurities, a process that should be repeated at least three times. In addition, several researchers have first rewashed the precipitate with ethanol and later washed it in distilled water several times [68,69]. The obtained wet precipitates are normally subjected to drying in an oven at a temperature between 60 and 70 °C for several hours or overnight to produce powdered ZnO NPs [3]. The dried sample can be calcined subsequently or kept at room temperature in an airtight container for future analysis and use. The purification of biogenic nanoparticles is a crucial step that must be carefully conducted; if not, the quality and purity of the synthesized nanoparticles will be compromised.

### 2.3. Calcination

Calcination, often called annealing, can eliminate and fix defects or impurities, activate the dopant atom, and enhance ZnO NPs’ electrical and optical properties [70]. Depending on the intended usage, these features may alter in different ways [1]. Calcination temperature greatly influences the size and quality of ZnO NPs, which are hexagonal wurtzite structures. The report by Karam and Abdulrahman [1] highlighted that an increase in calcination temperature from 150 to 450 °C resulted in larger particles (35.202 to 43.30 nm). It can be shown that varying the calcination temperature significantly affects the interplanar distance of biosynthesized ZnO NPs.

### 2.4. Phytochemical Screening

To identify the phytochemical compounds responsible for the stability and reduction of ZnO NPs, Liquid Chromatography Mass Spectrometry (LCMS), high-performance liquid chromatography (HPLC), and Gas Chromatography (GC) are crucial analytical tools commonly employed for this purpose. LCMS, a powerful technique, provides both qualitative and quantitative information about phytochemical compounds. HPLC, on the other hand, is instrumental in separating, identifying, and quantifying each component in a mixture. Gas Chromatography (GC) is particularly useful for analyzing volatile compounds. Together, these analytical methods enable the identification of phytochemical compounds involved in the reduction of metallic salts to nanoparticles, as well as those acting as stabilizing or capping agents on the surface of the synthesized nanoparticles [71,72].

A wide variety of bioactive substances, such as terpenoids, flavonoids, and polyphenols with reducing abilities, are found in plant extracts. These substances can function as stabilizing and reducing agents while synthesizing nanoparticles. They play a significant role in the reduction process by actively transferring electrons to the metal ions present in the precursor solution. This electron donation supports and accelerates the formation of nanoparticles, contributing to the overall efficiency of the process [73]. Analytical instruments can identify the phytochemical compounds that stabilize the synthesized nanoparticles [10,68,74]. These biomolecules attach themselves to the nanoparticle surface, stabilizing and preventing them from aggregating. It is also important to acknowledge that the size, shape, and surface properties of the generated nanoparticles are significantly influenced by the capping agents. As an essential component of nanoparticle synthesis, these capping agents control the final characteristics of the nanoparticles, including their stability, chemical reactivity, and interactions with other materials [55].

### 2.5. Physicochemical Characterization of Nanoparticles

Choosing a suitable technique for characterizing nanoparticles from the options available for determining their size, shape, aggregation, chemistry, and other characteristics is crucial because while fabricating nanoparticles, various issues arise regarding the characterization [25]. It is very important to determine the characteristics of any synthesized nanoparticles to understand their behavior and create new materials with certain qualities; thus, characterization techniques are employed to gather data on the chemical, physical, mechanical, and electrical characteristics of materials. Some of the common analytical instruments used for characterizing biogenic metallic nanoparticles are discussed below.

#### 2.5.1. Ultraviolet–Visible (UV–Vis) Spectrophotometry

**Figure 4:**
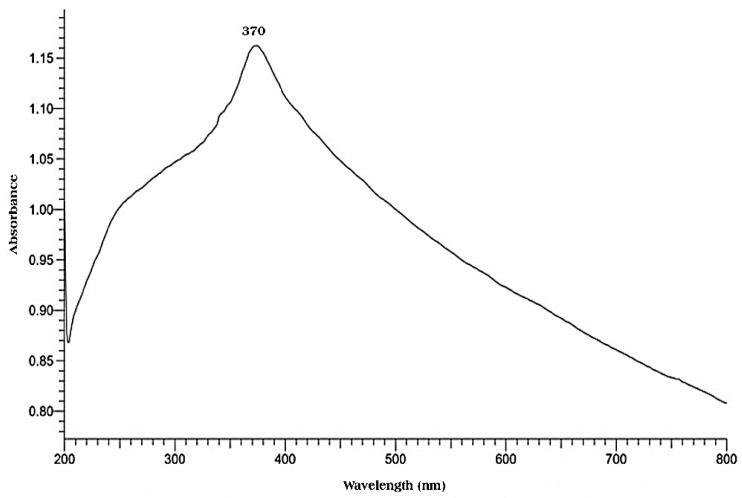
UV–Vis spectrum of ZnO NPs biosynthesized using an aqueous leaf extract *Pelargonium odoratissimum* (L.). Figure reuse from [27].

Plant extracts produce nanoparticles with varying forms, sizes, and crystallinity due to their unique reactions with metal ions [75]. The investigation observed a notable change in the colour of the metal ion solution from clear to pale brown, indicating the formation of smaller particles. This alteration can be attributed to the phytochemicals present in the plant extract, which possess the capability to convert metal ions into metal nanoparticles. By functioning as a stabilizing and reducing agent, the plant extract plays a crucial role in this process. Furthermore, the advancement of the reaction is carefully monitored and analyzed using UV–Vis spectroscopy, allowing for a comprehensive understanding of the dynamics involved in the conversion of metal ions to metal nanoparticles [76]. In the UV–visible spectroscopic spectrum, peak absorption is associated with surface plasmon resonance (SPR), which reflects metal ion reduction and nanoparticle development by interacting electromagnetic waves with the electron conduction band oscillation (Figure 4) [77].

The plant extract is a complex mixture containing tannins, phenolic acids, flavonoids, and essential oils. These components are believed to have significant potential as bio-reducing and stabilizing agents. This is attributed to the presence of multiple hydroxyl (-OH) groups, which can play a key role in the synthesizing of nanoparticles. Based on qualitative evidence, band-gap energy is shown to decrease with increasing calcination temperature [78]. The relationship between the narrowing band gap in ZnO nanoparticles and the presence of oxygen vacancies is noteworthy. It is suggested that the oxygen vacancy in these nanoparticles results from the disordered crystal structure caused by exposure to high calcination temperatures [79]. This finding underscores the importance of understanding the impact of synthesis conditions on the properties of ZnO nanoparticles. For example, Al-Askar et al. [10] reported that surface plasmon resonance (SPR) for biosynthesized ZnO NPs using *Pluchea indica* was recorded at 360 nm after 24 h. In another work conducted by Mbenga et al. [67], the absorption spectra of biosynthesized ZnO NPs using an extract of *Tulbaghia violacea* displayed the highest peak at 273 nm. Also, the biosynthesis of *Spirulina platensis*-mediated ZnO NPs was confirmed using a UV–Vis absorbance peak at 372 nm [80].

#### 2.5.2. Fourier Transform Infrared (FT-IR) Spectroscopy 

The infrared absorption and molecular vibrations of NPs are examined using FT-IR spectroscopy. The analytical method can offer comprehensive information about the exact chemical composition and the intricate interactions between the nanoparticles (NPs) and the molecules in their vicinity. Additionally, it can precisely identify the specific functional groups that are present on the surface of the nanoparticles [55]. Metal ions can be reduced in size to a nanometer by reacting with several functional groups found in plant phytoconstituents, including hydroxyl, carboxyl, and amine [61,81]. The reduction of metal ions into nanoparticles is thought to be caused by the -OH group found in flavonoids. Furthermore, these substances are crucial in the process of capping nanoparticles, ensuring their stability and biocompatibility and enabling the biological reduction of ions to nanoscale levels. This is essential for the functional and biomedical applications of nanoparticles in various fields [82]. FT-IR spectra of ZnO NPs may be obtained by absorbing electromagnetic radiation at 400–4000 cm^−1^. ZnO NPs exhibit a remarkable ability to effectively absorb electromagnetic waves spanning a wide spectrum of frequencies and intensities. This characteristic enables precise and detailed characterization of specific functional groups and intricate chemical structures, providing valuable insights into their properties and behavior [17].

Plant extracts include a wide range of phytochemicals that serve as functional groups for metal reduction. The most common are flavonoids, which may be found in all parts of plants and have lower molecular weights. The flavonoid content of plants is an important consideration when employing them for biogenic synthesis. According to Shafey [83], flavonoids stabilize nanoparticles and minimize their toxicity. Enzymes, vitamins, and phenolic compounds are essential phytochemicals that contribute to green synthesis. Recent pharmacological studies have extensively demonstrated that primary active constituents, such as alkaloids (such as caffeine and morphine), flavonoids (such as quercetin and catechins), and phenolic acids (such as gallic acid and caffeic acid), possess a wide range of beneficial properties. These properties include powerful antioxidant effects that help combat oxidative stress and reduce the risk of chronic diseases. Additionally, these constituents have shown potential in managing diabetes, combating various pathogens, and exhibiting promising anticancer activities [3].

**Figure 5:**
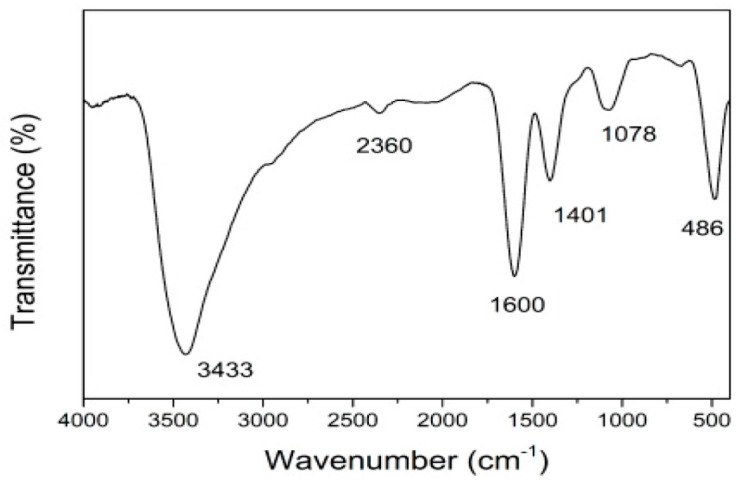
FT-IR spectra of ZnO NPs synthesized using aqueous extracts of *Hibiscus cannabinus* L. Figure reuse from [31].

In a single procedure, phenolic molecules, sterols, and alkaloids (nicotine) function as reducing agents, capping and stabilizing Zn metal ions to ZnO NPs. An indication of the ZnO nanostructure’s stretching was found by the FT-IR spectrum at 523 cm^−1^ [84]. Maher et al. [3] affirm that the Zn-O stretching vibration is what produces the 400–600 cm^−1^ peak (Figure 5). Furthermore, *Pluchea indica*-mediated ZnO NPs’ FT-IR measurement showed a significant signal at 432.05 cm^−1^, suggesting the presence of a Zn-O bond [85]. The Zn-O vibrations at 566 cm^−1^ were validated by Khan et al. [86].

#### 2.5.3. X-ray Diffraction (XRD) Analysis 

**Figure 6:**
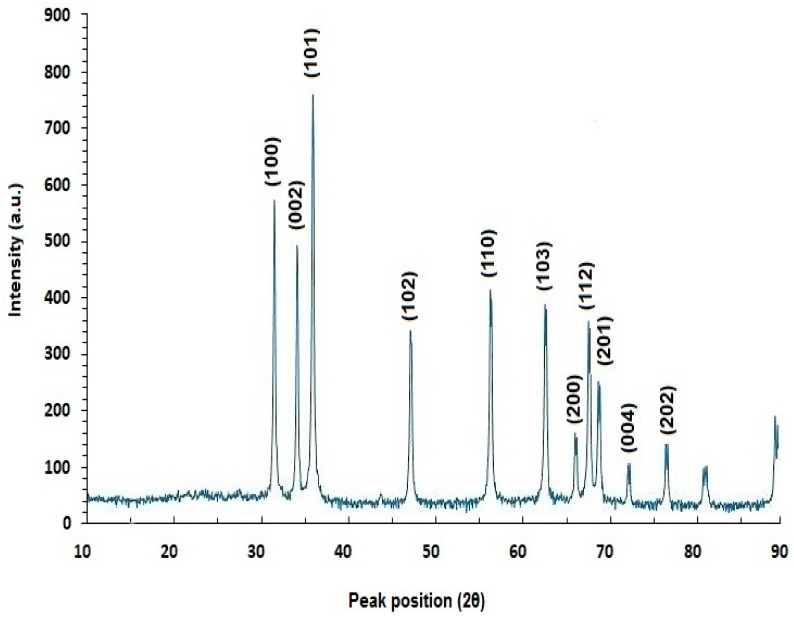
XRD patterns of green-synthesized ZnO NPs using *Phlomis* leaf extract. Figure reuse from [88].

XRD is a widely employed technique to analyze the structural properties of materials. For instance, in the context of ZnO samples, XRD can be employed to verify the sample’s purity and characterize its hexagonal wurtzite structure. This method is particularly beneficial in nanostructure research as it offers detailed insights into the underlying structure of materials. The breadth and shape of reflections obtained from XRD analysis can provide comprehensive information about various factors, including crystallite sizes, which are crucial for understanding the material’s properties and behavior [87]. Several scientists have utilized X-ray diffraction (XRD) to analyze and characterize ZnO NPs, with findings documented in various studies (Figure 6). Amin et al. [15] and El-Fallal et al. [33] have contributed to this area of study. Naiel et al. [44] reported that the XRD pattern of ZnO NPs mediated by *Limonium pruinosum* exhibited diffraction peaks at approximately 31.45°, 34.66°, 36.26°, 47.48°, 56.29°, 62.7°, and 68.31°. Similarly, Maher et al. [3] observed four main peaks at 2θ values of 31.8°, 34.5°, 36.27°, 47.5°, 56.7°, 62.8°, and 67.5° in their study. Additionally, Ihsan et al. [20] reported various peaks at 2θ values of 32.76°, 34.43°, 36.24°, 47.59°, 57.61°, 63.87°, and 68.9° from their XRD patterns of biosynthesized ZnO NPs. Furthermore, Alyamani et al. [88] observed several diffraction peaks at 2θ = 31.8341°, 34.4911°, 36.321°, 47.6034°, 56.6643°, 62.9192°, 66.4384°, 68.0045°, 69.1421°, 72.6285°, and 77.0281°, in their investigation of *Phlomis* leaf-mediated ZnO NPs.

#### 2.5.4. Scanning Electron Microscopy (SEM)

**Figure 7:**
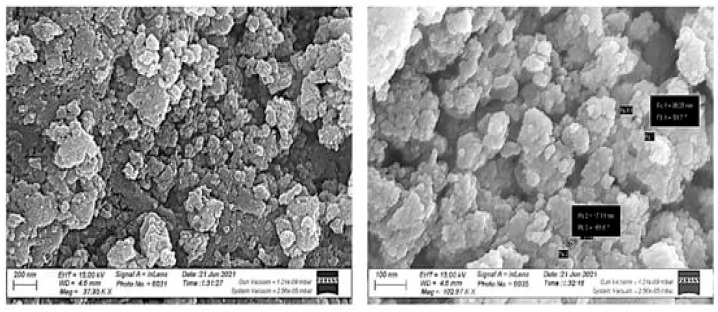
SEM image of ZnO NPs synthesized using aqueous leaf extract of *Pelargonium odoratissimum* (L.). Figure reuse from [27].

Using an electron beam, SEM provides high-resolution surface imaging that may be utilized to understand an object’s nano- and micro-scale characteristics [89]. SEM images provide a larger field of view and a higher magnification, which makes them useful for assessing the topology of ZnO NP surfaces [90]. For morphological identification, this technique depends on visual assessment [91]. When ZnO NPs are exposed to electron beams, the detector generates and records signals. The shape, orientation, and crystalline structure of the ZnO NPs may be inferred from the signal [92]. According to the study by Abdelbaky et al. [27], the ZnO NPs synthesized using *Pelargonium odoratissimum* had excellent distribution and spherical and hexagonal shapes (Figure 7). Mbenga et al. [67] observed that the ZnO NPs mediated by *Tulbaghia violacea* were spherical and closely packed. Similarly, Ahmed and Othman [80] reported that SEM images indicated that ZnO NPs exhibited excellent dispersion and were hexagonal, with some rough aggregations. The SEM of ZnO NPs prepared from *Beta vulgaris* showed that the particles are almost spherical and clustered together to form a sponge-like accumulation of particles [93]. Faisal et al. [94] revealed that the ZnO NPs synthesized in the aqueous fruit extract of *Myristica fragrans* had particles that were strongly agglomerated and had a semispherical shape. This makes it abundantly evident that the particles are homogenous and that their functions depend significantly on their homogeneity. ZnO NPs synthesized from *Pistacia lentiscus* L. exhibited an aggregated, dried cotton-like appearance [51]. The majority of these ZnO NPs synthesized using *Cocos nucifera* leaf extract exhibited an aggregated, dried cotton-like appearance [21]. The hydrogen bonding and electrostatic interactions between bioorganic capping molecules and the NPs have caused them to accumulate together. The SEM images of ZnO NPs showed that they are not in direct contact with each other, indicating the stability of NPs by capping agents. Amin et al. [15] observed that ZnO NPs synthesized from *Lentinula edodes* demonstrated that the bulk of components are spherical, and they aggregate into larger particles with unclear geometry. The SEM and size distribution of ZnO NPs show a restricted range of particle sizes, with a diameter of approximately 200 nm. The findings of Alghamdi et al. [5] found that the *Celosia argentea*-mediated ZnO NPs showed a non-uniform spherical shape and an accumulation of the formed semispherical particles. It was reported that using biomolecules as reducing agents increases particle aggregation due to the electrostatic interaction between metal ions and biomolecules. Furthermore, SEM images of *Pelargonium odoratissimum* (L.)-mediated ZnO NPs showed that they were spherical and hexagonal in morphology, with good distribution and an average size of 21.6 nm [27]. ZnO NPs prepared from *Riobotrya japonica* leaf extract showed irregular spherical shapes and most likely to be hexagonal shapes that agglomerated into large network structures [34].

#### 2.5.5. Energy-Dispersive X-ray (EDX)

**Figure 8:**
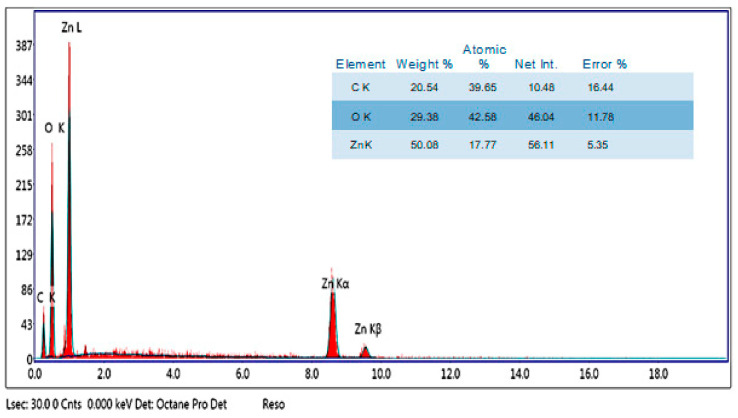
EDX spectrum showing elemental compositions of biosynthesized ZnO NPs using an extract of *Pluchea indica.* Figure reuse from [10].

The analytical technique known as EDX is normally used to determine the purity and chemical composition of NPs [93]. In EDX, NPs are subjected to an intense electron beam bombardment, and the X-rays they release are monitored. Since the rays are produced based on the characteristics of the elements, the atomic structure of each element results in unique peaks in the X-ray spectrum [17]. In a study conducted by Vijayakumar et al. [92], the composition of ZnO NPs synthesized using *Plectranthus amboinicus* extract was analyzed using EDX spectroscopy. The results revealed that the ZnO NPs contained 75.12% zinc, 23.55% oxygen, and a small peak that was attributed to the presence of bound bio-compounds from the *Plectranthus amboinicus* extract. This detailed chemical analysis provided valuable insights into the elemental composition and potential functional groups associated with the synthesized ZnO NPs. Furthermore, Al-Askar et al. [10] studied ZnO NPs synthesized using *Pluchea indica*. The analysis showed that these nanoparticles comprised 20.5% carbon, 29.3% oxygen, and 50% zinc (Figure 8). This comprehensive characterization shed light on the specific elemental ratios and constituents present in the ZnO NPs synthesized using *Pluchea indica*, providing a deeper understanding of their chemical structure and potential properties. In another investigation, Gamedze et al. [68] explored the synthesis of ZnO NPs using *Mucuna pruriens* (utilis). The results demonstrated that these nanoparticles primarily consisted of zinc and oxygen, highlighting the high purity of the synthesized ZnO NPs. This thorough analysis of the elemental composition and purity of ZnO NPs synthesized using *Mucuna pruriens* (utilis) has contributed to the growing body of knowledge regarding diverse approaches to nanoparticle synthesis and their resulting properties. In another study conducted by Naiel et al. [44], the presence of zinc in its oxide state and the purity of the produced ZnO NPs were verified using EDX results. At around 1 keV, 8.6 keV, and 9.6 keV, strong emission peaks for zinc were found. Carbon and oxygen emission maxima at 0.3 and 0.5 keV may result from plant biomass used in phyto-synthesis. Zn and O were confirmed to be present in ZnO NPs produced from *Pistacia lentiscus* L., with approximate weight percentages of 38.15% for Zn and 52.13% for O [51]. Rahman et al. [21] observed the presence of a large percentage of Zn and O as an indication of ZnO formation in the biogenic ZnO NPs synthesized from *Cocos nucifera* leaf extract. Also, the EDX analysis of *Pelargonium odoratissimum* (L.)-mediated ZnO NPs revealed that they contain Zn (80.71%) and O (19.29%) by weight [27]. Elemental mapping analysis of ZnO NPs prepared from *Erminalia catappa* fruit pericarp revealed that the quantities of Zn and O were 77.67 and 22.33% when measured in weight percentage [43]. The existence of Zn and O in the ZnO NPs produced from *Pistacia lentiscus* L. is confirmed via the EDX analysis, which shows approximate weight percentages of approximately 38.15% for Zn and 52.13% for O [51].

#### 2.5.6. Thermogravimetric Analysis (TGA)

**Figure 9:**
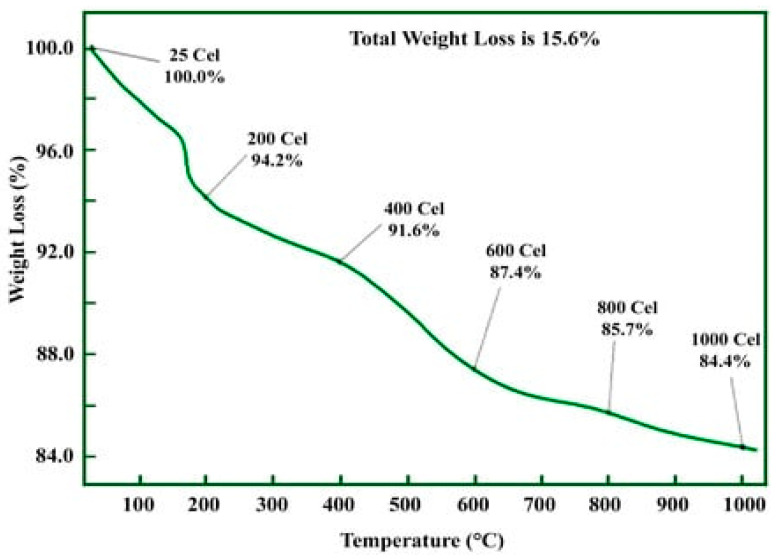
TGA pattern of ZnO NPs synthesized using an aqueous fruit extract from *Crotalaria verrucosa.* Figure reuse from [97].

In this analysis, the samples are subjected to high temperatures of up to 900 °C to determine the thermal decomposition of the biosynthesized ZnO NPs. The TGA spectra of ZnO NPs show that the sample decomposes significantly as temperature increases. For instance, Faisal et al. [94] observed that the initial weight loss of the sample was attributed to the presence of both ethanol and water, as supported by findings from Pomastowski et al. [95] and Yusof et al. [96]. Yusof et al. [96] further documented a 2.78% weight loss at a temperature of 237 °C, indicating the potential elimination of coordinated water molecules. Additionally, a total weight loss of 2.79% at 660 °C was observed, suggesting the possible involvement of the hydroxide group on the ZnO NPs and the breakdown of an organic component. Faisal et al. [94] reported that the *Myristica fragrans*-mediated ZnO NPs completely decomposed when subjected to 600 °C. This observation could be due to the presence of volatile compounds in the sample originating from the plant extract used in the synthesis, and no further noticeable changes were noted (Figure 9). Moreover, Sana et al. [97] reported that the loss of humidity in *Crotalaria verrucosa*-mediated ZnO NPs was shown by a weight decrease at about 120 °C. The sample’s minimal number of organic components was eliminated and decayed between ~340 and 550 °C, which is when the weight loss was noticed. This phenomenon was attributed to the presence of diverse volatile components from the extract, which acted as capping agents for the nanoparticles. Interestingly, the biogenic ZnO NPs retained a high weight at 1000 °C, suggesting the sample’s remarkable level of thermal stability [30].

#### 2.5.7. Dynamics Light Scattering (DLS)

**Figure 10:**
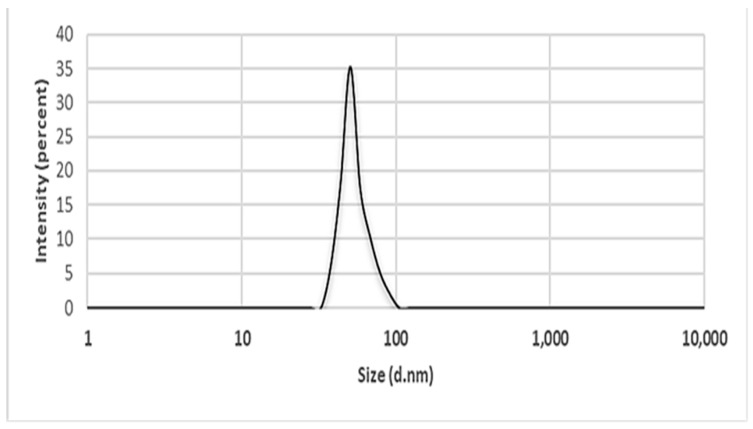
Particle size distribution of ZnO NPs synthesized using *Pluchea indica* leaf extract measured using DLS. Figure reuse from [10].

DLS is an advanced, non-invasive technique that accurately determines the size distribution of small particles or polymers in a solution. This method measures the scattering of laser light caused by the Brownian motion of particles, providing detailed information about their size and distribution. By applying the Stokes–Einstein relationship, DLS enables precise quantification of particle size, making it a valuable tool in various scientific and industrial applications. By analyzing individual scattering events, DLS sheds light on the dynamic characteristics of soft materials and is an effective method for investigating particle diffusion in a range of settings [98,99]. ZnO NPs are usually measured into the cuvette after being diluted in deionized water and vigorously vortexed for five minutes. This analytical tool has been employed in several studies in the literature [5,43,95,100]. For example, the study by Alyamani et al. [88] observed a small aggregation with an average hydrodynamic diameter of 165 ± 3.0 nm. According to the report by Al-Askar et al. [10], the average particle size of the ZnO NPs synthesized using *Pluchea indica* extract was 50.7 nm (Figure 10).

#### 2.5.8. Zeta Potential

The zeta potential magnitude reveals information about the surface charge of ZnO NPs and the potential stability of nanoparticles in colloidal suspension [101]. Nanoparticles’ stability, pharmacokinetics, and bioactivity are all influenced by their surface charge. A high zeta potential value suggests higher electrostatic repulsion between nanoparticles, resulting in improved colloidal stability [102]. According to research, particles with zeta potential values less than −30 mV or larger than +30 mV tend to form stable suspension due to comparable charges repelling and preventing aggregation [103]. Nanoparticles with low zeta potential values frequently agglomerate because of Van der Waals forces between individual particles [104]. The possible capping of bioorganic components present in the plant extracts might be the cause of the negative zeta potential value displayed by the zinc oxide nanoparticles. The development of stable ZnO NPs free from agglomeration is facilitated by high negative zeta potential values, which indicate electrostatic repulsion between the particles.

**Figure 11:**
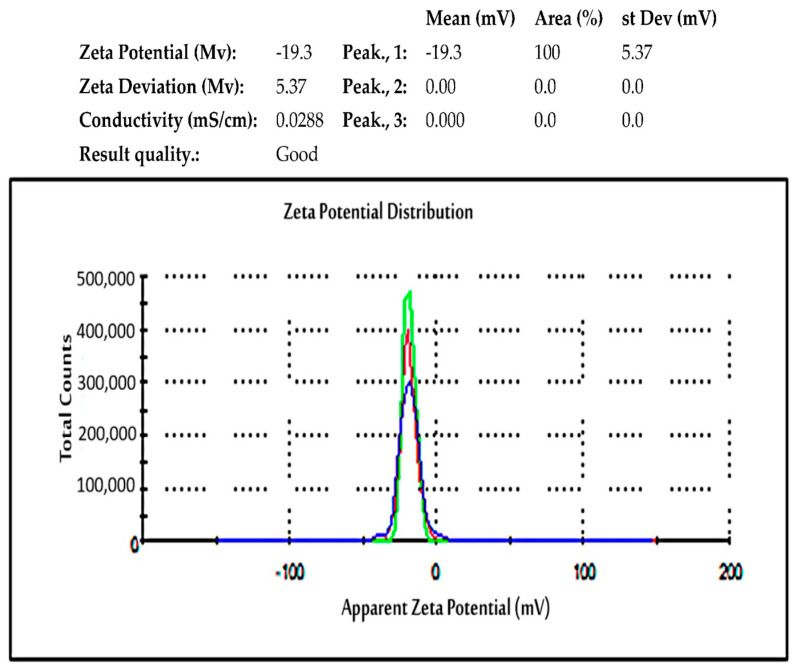
Zeta potential of ZnO NPs produced using an extract of *P. odoratissimum.* Figure reuse from [27].

For instance, *Tectona grandis*-mediated ZnO NPs have a zeta potential of 25 mV [105]. This value suggests the stability of the generated ZnO NPs. The authors believed the unfavorable outcome is caused by the reducing and stabilizing components, such as phenolic compounds, found in *T. grandis* extract. According to the report by Abdelbaky et al. [27], ZnO NPs obtained from the extract of *Pelargonium odoratissimum* (L.) exhibited a zeta potential of −19.3 mV, which suggests their possible stability [105] (Figure 11). This indicates that the phenolic and flavonoid components of the leaf extract function as reducing agents and are responsible for the ZnO NPs’ negative charge potential. It also indicates that the substance that was produced has significant electrostatic effects.

#### 2.5.9. Transmission Electron Microscopy (TEM)

The size and crystalline characteristics of the produced nanoparticles were identified via TEM examination. ZnO NPs’ TEM images demonstrated their hexagonal shape and minimal thickness fluctuation, which is consistent with the SEM findings. Their physiochemical characteristics greatly influence the solubility and absorption of nanoparticles. Small-sized nanoparticles have higher absorption and uniform distribution and are often removed via urine [106]. Size influences the overall pharmacokinetic behavior of nanoparticles. The sizes of ZnO NPs ranging from 1–100 nm synthesized using plant extract have been well documented by several researchers in previous studies [74,107,108].

**Figure 12:**
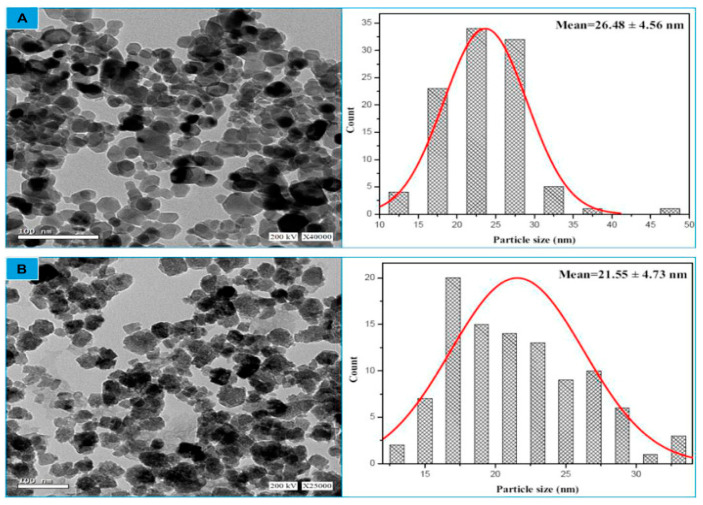
A representative of TEM image of ZnO NPs synthesized using plant extract. Figure reuse from [5]. (**A**) chemical ZnO NPs particle size distribution (**B**) biogenic ZnO NPs particle size distribution.

For instance, Alghamdi et al. [5] highlighted that the Celosia argentea-mediated ZnO NPs showed a non-uniform spherical shape (Figure 12). Also, Naiel et al. [44] noted that hexagonal/cubic forms of green-produced ZnO NPs, with an average size of approximately 41 nm, were visible in the TEM images of ZnO NPs prepared from an extract of *Limonium pruinosum* L. Chaz. According to Khan et al. [86], ZnO NPs generated from strawberry waste extract were well-distributed, homogenous, mostly spherical, and well-crystalline, with an average size of 50 nm. For example, Gamedze et al. [68] reported an average size of 30.50 nm for ZnO NPs synthesized using an aqueous extract of *Mucuna pruriens* (utilis), whereas Chan et al. [109] reported an average size of 14.21 nm for ZnO NPs synthesized from the leaf extract of mangosteen (*Garcinia mangostana* L.). Also, a study conducted by Mbenga et al. [67] documented an average size of 45.26 nm for ZnO NPs synthesized using an extract of *Tulbaghia violacea*. Naiel et al. [44] established that ZnO NPs synthesized using an aqueous extract of sea lavender, *Limonium pruinosum* (L.) Chaz., as a reducing agent, showed a hexagonal/cubic shape with an average size of ~ 41 nm in TEM analysis. Also, in another study, ZnO NPs synthesized from *Myristica fragrans* showed spherical- to hexagonal-shaped particles with a grain size of 35.5 nm [94]. The investigation by Mohamed et al. [12] revealed that ZnO NPs synthesized using fruit extracts of *Hyphaene thebaica* showed a quasi-spherical morphology on HR-TEM analysis. Also, *Pelargonium odoratissimum* (L.)-mediated ZnO NPs displayed hexagonal shapes with an average size of 34.12 nm in TEM analysis [27]. The study by Velsankar et al. [91] found that ZnO NPs prepared from *Echinochloa frumentacea* grain powder extract were hexagonal with definite edges and sizes ranging from 35 to 85 nm. Furthermore, TEM analysis of Mentha piperita-ZnO NPs revealed globular and oblong-shaped nanoparticles with sizes ranging from 15 to 27 nm. The even distribution and capping of biomolecules in the extract were demonstrated by the size and shape of the ZnO NPs. The investigation by Abduljabbar et al. [110] found that ZnO NPs prepared from *Euphorbia retusa* extract were spherical, trigonal, and tetragonal shapes.

## 3. Biocompatibility and Cytotoxicity Testing

Zinc plays a vital function in maintaining cell homeostasis and is a significant cofactor in various biological processes, making zinc oxide biocompatible. ZnO either participates in the body’s active nutritional processes or degrades fast. ZnO NPs are innately more cytotoxic to in vitro cancer cells compared to other metal nanoparticles. Although extracellular zinc oxide is innocuous, higher intracellular zinc oxide concentrations may indicate increased cytotoxicity due to zinc-mediated protein synthesis mismatch and oxidative stress [111]. The biocompatibility of ZnO NPs in blood was evaluated through hemolytic activity testing. Hemolysis consistently indicates a substance’s biological incompatibility since it depends on the direct or indirect adverse impact on the red blood cell membrane. Assessing a biomaterial’s safety in blood contact is one test used to determine its hemolytic potential [112]. Hemolysis occurs when red blood cells break down and release hemoglobin. For example, Neamah et al. [113] found that ZnO NPs generated from *Capparis spinosa* fruit extract had lower hemolytic activity compared to the control at dosages ranging from 7.5 to 120 μg/mL.

The cytotoxicity test is usually conducted using the 3-(4,5-Dimethylthiazol-2-yl)-2,5-diphenyltetrazolium bromide (MTT) assay [114]. For example, ZnO NPs produced using fruit extract from *Capparis spinosa* L. showed no cytotoxic activity against normal cells (a healthy L929 fibroblast cell line) in a dose-dependent manner [113]. Majeed et al. [115] recorded less cytotoxic activity of ZnO NPs synthesized from *Artocarpus heterophyllus* against Vero cells. According to Abduljabbar et al. [110], ZnO NPs produced with *Euphorbia retusa* extract demonstrated moderate activity against hepatocellular carcinoma (HePG-2) and breast cancer (MCF-7) cells, with IC_50_ values of 31.75 and 30.05 µg/mL, respectively, and a cytotoxic effect on the human prostate (PC3) cell line (IC_50_ = 16.04 µg/mL). In a different study, ZnO NPs produced from pomegranate peel considerably reduced the viability of colon cancer cell lines (HCT116) and MCF7 in a dose-dependent manner. By reducing cell viability to 0% at 50 µg/mL, ZnO NPs can eliminate MCF7. Neamah et al. [113] reported that ZnO NPs from *Capparis spinosa* demonstrated a low cytotoxic effect on L929 normal fibroblast cells.

Al-darwesh et al. [17] highlighted that reducing the rate of dissolution of ZnO nanoparticles by adding Fe atoms or coating their surface with a protective layer might increase their biocompatibility. Therefore, efforts have been made to improve ZnO NPs’ suitability for use in clinical settings by covering their surface with biocompatible macromolecules such as polyethylene glycol (PEG), chitosan, and poly(lactic) acid. A different idea is to employ clinically confirmed biodegradable and biocompatible materials to create ZnO nanoplatforms. Biocompatible polymers such as liposomes, aptamers, and dendrimers have been approved for use in clinical settings.

## 4. Antibacterial Activity of ZnO NPs

Despite enormous progress in the prevention and treatment of infectious diseases, which are increasing daily, infections continue to be one of the leading causes of mortality worldwide [116,117]. Microbial resistance has increased because of antibiotic overuse in recent decades. Antimicrobial resistance was one of the top ten worldwide public health problems identified by the World Health Organization in 2021. In 2019, six important bacterial infections, including methicillin-resistant *Staphylococcus aureus* and third-generation cephalosporin-resistant *Escherichia coli*, were linked to the highest resistance-related mortality rates. Most pathogenic microbes can become resistant to at least some antimicrobial agents [118]. Several factors contribute to antibiotic resistance in bacteria, including active excretion of antibiotics from cells, changes in antibiotic targets [119,120], and prevention of drug penetration into a cell [121,122].

The number of newly approved antibacterial drugs with unique modes of action has decreased recently [123]. In addition to having negative side effects, antibiotic misuse is a major factor in the rise of bacteria that are resistant to antibiotics [124]. Moreover, bacteria that form biofilms and cause persistent infections have become increasingly resistant to human defense systems and antibiotic treatment [125]. Therefore, developing new antibiotics that are exceptionally effective, have low toxicity, and have fewer serious side effects seems crucial for enhancing infection control [74]. The use of drug delivery systems and nanoscience to develop novel pharmaceutical technologies seems to be a potential way to address this urgent demand for action against microbial resistance, especially considering recent advances, particularly in medicinal biology. The primary reason for the antibacterial activity of biogenic ZnO NPs against different strains of bacteria is their physicochemical features, which include their size, shape, surface chemistry, and high surface-to-volume ratio. This increased contact may strengthen the antibacterial activity of the NPs by facilitating easier penetration of the bacterial cell wall and disrupting cellular processes [5,126]. Several medicinal plants have been explored for synthesizing ZnO NPs in previous studies, and some of the investigations are summarized in Table 1. Biogenic ZnO NPs are safer and have minimal cytotoxic effects compared to conventional antibiotics, in which their usage is associated with several cytotoxic effects. The cost of production of biogenic zinc oxide nanoparticles is cheaper compared to the cost of conventional antibiotics. Biogenic zinc oxide nanoparticles have multiple target sites for action compared to antibiotics specifically designed to target one site. 

## 5. Antibacterial Assays

The disk diffusion method is generally employed to evaluate the in vitro antibacterial activity of biogenic ZnO NPs against different pathogenic bacteria in the literature [20,21]. In this method, Mueller–Hinton broth agar is used to form an agar medium for culturing bacteria, and after the bacteria is resuscitated in LB broth or nutrient broth to reach the log phase of growth, the suspension is standardized by adjusting to 0.5 McFarland standards (equivalent to 1–2 × 10^8^ CFU/mL). Subsequently, the bacterial suspensions are inoculated onto a Mueller–Hinton agar and incubated at 37 °C for 24 h. Thereafter, wells with a diameter of 6 mm are created in the agar using a sterile cork borer. Different concentrations of ZnO NPs are then applied to the wells and incubated overnight at 37 °C for 24–72 h. Afterward, the zone of inhibition is measured in mm using a standard metric ruler. Antibiotic discs are usually used as positive controls, while DMSO always serves as the negative control.

The broth microdilution method is another method to determine the antibacterial activity of biogenic ZnO NPs [51]. In this method, distinct bacterial colonies are inoculated in Mueller Hinton broth and incubated overnight at 37 °C for 24 h. After that, the bacterial suspension is standardized to the 0.5 McFarland standard using sterile saline water; 100 µL of the suspensions is added to each well, and ZnO NPs are prepared and added to the first well in the microtiter plate, and two-fold serial dilutions are made subsequently. Later, microtitre plates are sealed using parafilm and incubated at 37 °C for 24 h. Next, the incubated microtitre plate wells are filled with 0.01% resazurin sodium salt indicator and incubated for 2 h at 37 °C. This salt reacts with viable bacteria to produce colour changes. A purple or blue colour change indicates bacterial inhibition, while colourless or pink colour changes indicate viable bacteria that can reduce resazurim sodium salt to resorufin. Therefore, the minimum inhibitory concentration (MIC) is the lowest concentration of ZnO NPs, which can prevent bacteria from growing visibly after overnight incubation. The minimum bactericidal concentration (MBC) can be established by streaking 10 μL of the bacterial suspension from the well, which is greater or equal to the lowest MIC on the Mueller Hinton agar, and incubating for 24 h. Subsequently, the plates are investigated for possible bacterial growth, and the minimum concentration of ZnO NPs with no visible growth indicates the minimum bactericidal concentration (MBC).

## 6. Possible Mechanism of Action of ZnO NPs

**Figure 13:**
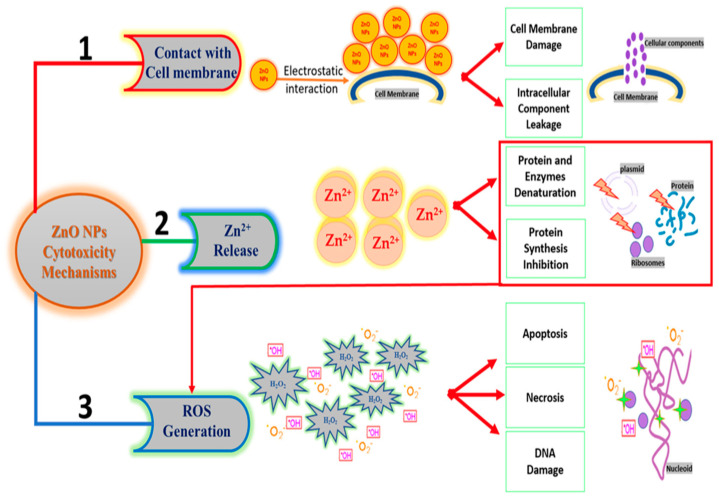
Cytotoxicity mechanisms of biogenic ZnO NPs [26].

The surface area of membranes is increased by nanoparticles, which improves their antibacterial action [172]. Reactive hydroxyl radicals, which can cause irreversible damage by oxidizing proteins and damaging RNA and DNA, are introduced by the nanoparticles after interacting with compounds such as phosphorus and sulfur [173,174,175]. This ultimately changes and destroys the helical structure of nucleic acids. Toxic metal ions are released during the breakdown of proton efflux bombs, influencing the respiratory system’s permeability and infectious membranes. Furthermore, nanoparticles have a bactericidal or inhibitory effect because they inhibit cell membrane enzymes due to their attraction to the membrane [81,176,177]. This helps NPs that are electrostatically attached to membrane-based plasma reductases oxidize. Once within the cytosol, these ions penetrate lipid layers, generating oxygen species such as O_2_, which then transforms into H_2_O_2_, leading to the oxidation of proteins and lipids (Figure 13) [178]. According to Jayaseelan et al. [179], there is yet another theory that suggests ZnO NPs are lethal because they attach themselves to bacterial cell membranes and build up inside the cytoplasm, damaging the membrane’s integrity and allowing its contents to leak out, ultimately resulting in cell death. The surface area and, thus, the antibacterial activity of nanoparticles increase with decreasing NP size. Bacterial cell membranes are typically nanometers in size. According to Naseer et al. [127], nanoparticles smaller than cell membrane pores can penetrate the cell membrane barrier and prevent bacterial growth (Figure 13).

Improving drug delivery for treating illnesses is becoming increasingly popular [180]. Thus, candidates such as biogenic metal-based nanoparticles with enhanced pharmacokinetics and biodistribution have a rare potential because of this interest [181]. According to Sutradhar and Amin [182], metallic nanoparticles inspired by nature are a novel class of nanomedicines that are designed to mimic natural circulation cells. It has been reported that these substances may increase the time a drug remains in blood circulation and enhance the drug’s distribution to cells and tissues. According to Sutradhar and Amin [182], the use of nanotechnology in targeted treatment of illnesses that frequently have less severe side effects has the potential to positively influence clinical practice and advance lifesaving methods. Nevertheless, throughout clinical trials, their immunogenicity, scaling up, and characterization continue to be significant obstacles [183]. In addition to scaling-up issues, government restrictions and the general cost-effectiveness of nanomedicines relative to currently existing chemotherapies are significant barriers to their development.

### How Biofilms Mediate Antimicrobial Resistance

The complex antimicrobial resistance (AMR) process is mostly driven by biofilms [184]. Biofilm-forming bacteria can exhibit a 10 to 1000-fold increase in antibiotic resistance compared to corresponding bacteria in a planktonic environment [185]. One of the most significant advantages of the biofilm environment for bacteria is the close connection of several species to one another [186]. This facilitates the transmission of mobile genetic elements and bacterial communication through techniques like quorum sensing [187]. The biofilm environment promotes plasmid stability and facilitates the easier transmission of resistance information among organisms [188]. To make matters worse, many of the transposable DNA elements transmitted by bacteria encode biofilm-promoting proteins, which contribute to the biofilm’s persistence and infection in patients [189].

Surgeons, doctors, and other healthcare workers may find it challenging to diagnose infections driven on by biofilms on medical equipment, even though biofilm-associated diseases can pose a persistent and sometimes fatal risk to patients [190]. It frequently entails sampling the surfaces of medical devices, which may involve invasive techniques like aspiration or biopsy (removal of the device by surgery). Blood cultures, bodily fluids, or other tissues linked to the illness, however, could also be useful. Sampling is followed by standard microbiological culture for identification and antibiotic susceptibility testing. Unfortunately, fastidious (difficult to cultivate) or uncommon microbial cultures frequently make this challenging. Furthermore, a polymicrobial biofilm could exist. Identifying the appropriate antibiotic treatment regimen can be tricky when dealing with mixed cultures, particularly if the drug is targeting a dominating bacterium that is growing faster than the polymicrobial community. As antimicrobial stewardship and infection prevention programs evolve, it will become increasingly important to comprehend the threats posed by biofilms and how limiting the transmission and acquisition of biofilm-forming organisms may negatively impact the emergence of antimicrobial resistance (AMR) [191].

**Figure 14:**
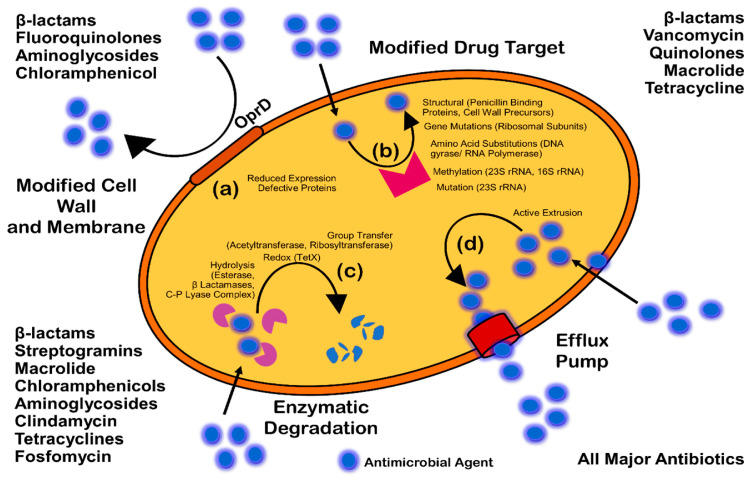
Different mechanisms of antibacterial resistance [192].

Both antibiotic tolerance and resistance can occur in biofilms. Microorganisms develop resistance mechanisms against antibiotics through horizontal gene transfer (HGT) within biofilm extracellular polymer systems (EPS), genetic mutation, or the acquisition of foreign genetic material that codes for resistant determinants [192]. Several mechanisms, including decreased permeability or access to antimicrobials, target alteration or mutation, and enzymatic destruction of antimicrobials via hydrolysis or chemical change, cause antimicrobial resistance (AMR) (Figure 14).

## 7. Conclusions, Challenges, and Future Prospects

Currently, efforts are focused on developing more environmentally friendly processes for producing ZnO NPs for different therapeutic purposes. Green-manufactured zinc oxide nanoparticles are highly valued for their affordability, biocompatibility, and reduced adverse environmental effects compared to their chemical and physical counterparts. ZnO NPs may be sustainably synthesized using a range of plant extracts, including phytochemicals such as tannins, alkaloids, polyphenols, terpenoids, and flavonoids. This process makes ZnO NPs more stable, and their distinct physicochemical properties may be examined. Several investigations have shown that the distinctive phytochemicals on the surfaces of ZnO nanoparticles produced using eco-friendly methods result in outstanding biological activity. ZnO NPs have exceptional antibacterial ability, making them extremely promising for treating diseases caused by drug-resistant bacteria. Furthermore, zinc is an essential trace element present in the human physiological system, and its solubility results in low toxicity and biodegradability, making it ecologically useful.

Green nanotechnology has many advantages, but it also presents several challenges. Scaling up is a major challenge; now, the only way to produce green nanomaterials is through laboratory-scale research, and it is difficult to translate these findings into large-scale production procedures. The main challenge is selecting the bioresource that will optimally produce the necessary nanoparticles. Separating synthesized nanoparticles from their fundamental biological constituents is another significant difficulty. The chemical composition of plant extracts can be significantly influenced by various factors, including plant species, growth environments, and the nature of the extraction solvent. This inconsistency hampers quality control and reproducibility, and it might result in differences in the synthesis process and properties of the nanoparticles produced. Plant extracts may include a range of bioactive chemicals that interact with nanoparticle formation. The presence of numerous chemicals may influence the kinetics and mechanics of nanoparticle growth, making it difficult to accurately manage the synthesis parameters. Also, green synthesis can involve relatively longer synthesis times and variability in nanoparticle properties due to the natural variability of plant extracts, which can affect the consistency and reproducibility of the nanoparticles produced.

Also, it is common practice to prepare plant aqueous extracts fresh for the synthesis of ZnO NPs, as aqueous extracts cannot be refrigerated due to fungal contamination. Therefore, to preserve the integrity of the physiochemical compounds in the plant extract, it would be beneficial for future research in this area to consider the use of lyophilized plant extract for ZnO NP synthesis. Several options are being investigated by researchers to address these challenges. To maximize ZnO NPs’ antibacterial activity and reduce cytotoxicity, one strategy is to optimize the synthesis and surface modification. Modifying ZnO NPs’ size, shape, and surface chemistry to enhance their interactions with bacteria and lessen their effects on mammalian cells might be one way to achieve this. More research is needed to understand how ZnO NPs work effectively and to determine their antibacterial effect on specific pathogens.

Furthermore, although ZnO NPs have the potential to improve human health, bringing these materials to the clinic presents several hurdles. One of the challenges is a prevalent misunderstanding about the biological effects and ZnO NP cytotoxicity. Disparities in the literature are most likely due to a lack of shared knowledge between biological scientists and materials scientists about each other’s limitations and capabilities. Nanoparticles are not always similar between batches and may differ in surface chemistry or size distribution, there is no common understanding between materials scientists and biological scientists regarding the synthesis of nanoparticles and the sensitivity of their biological applications. Likewise, the stability of nanoparticles is an important issue that could limit the clinical use of ZnO NPs, and this issue can be overcome by surface functionalization to improve their stability.

All these challenges need to be addressed to foster the industrial synthesis of plant-based nanoparticles that can meet the clinical demand for novel, safe, and cost-effective antibacterial agents that could be used to fight against multidrug-resistant bacteria, which pose a serious threat to public health.

## Figures and Tables

**Table 1:** List of some green synthesized ZnO NPs using plant extracts.

Plant	Part Used	Concentration of Salt	Average Size (nm)	Shape	Reaction Time	Reaction Temperature and pH	Test Bacteria	Reference
*Limonium pruinosum*	Whole plant	0.5 M zinc acetate dihydrate	~41 nm	Hexagonal/cubic	30 min	70 °C pH 8	*Bacillus subtilis* (ATCC 6633), *Staphylococcus aureus* (ATCC 6538), Gram negative bacteria (*Escherichia coli* (ATCC 8739), and *Enterobacter aeruginosa*	[44]
*Cassia fistula* and *Melia azadarach*	Leaves	0.01 M zinc acetate dihydrate	68.1 nm and 3.62 nm	Spherical	60 min	70 °C	*Escherichia coli* (*E. coli*) and *Staphylococcus aureus* (*S. aureus*)	[127]
*Cocos nucifera*	Leaves	0.05 M aqueous solution of Zn(NO_3_)_2_·6H_2_O	16.6 nm	Spherical/hexagonal	3 h	12	*Staphylococcus aureus* (cars-2), *Bacillus megaterium* (BTCC-18), and *Bacillus cereus* (carsgp-1)	[21]
*Myristica fragrans* (Jaiphal)	Fruit	zinc acetate dihydrate	41.23 nm	Flower-shaped	2 h	60 °C	*Klebsiella pneumoniae*, *Escherichia coli*, *Pseudomonas aeruginosa*, and *Staphylococcus aureus*	[94]
*Ziziphus mauritiana Lam*	Leaves	(Zn(NO_3_)_2_·6H_2_O)	63–83 nm	Spherical	-	60 °C	*Staphylococcus aureus* and *Escherichia coli*	[128]
*Viscum* *album*	Leaves	0.01 M zinc acetate dihydrate	13.5 nm	Quasi-spherical	30 min	70 °C	*Pseudomonas aeruginosa* (ATCC 10145), *Escherichia coli* (ATCC 10799), *Staphylococcus aureus* (ATCC 29213), and *Bacillus subtilis* (ATCC 11774)	[129]
*Pistacia lentiscus*	Leaves	0.1 mol/L (Zn(CH_3_COO)_2_ and 2H_2_O)	33.90 nm	Dried cotton-like appearance	30 min	78 °C	Staphylococcus aureus, Bacillus cereus, Escherichia coli, and Pseudomonas aeruginosa	[51]
*Cymbopogon citratus*	Leaves	(Zn(NO_3_)_2_ 6H_2_O)	20–24 nm	Hexagonal rod-like shape	5 min	Room temperature	*S. aureus* (MTCC 9760) and *E. coli* (MTCC 443)	[130]
*Clitoria ternatea*	Flower	Zn (NO_3_)_2_·6H_2_O	41 nm	Partially/roughly spherical	2 h	60 °C	*Escherichia coli* and *Staphylococcus aureus*	[131]
*Artemisia aucheri*	Leaves	1g in 15 mL of Zn(NO_3_)_2_	76 nm	Spherical and granular	20 min	Room temperature	*Escherichia coli* and *Staphylococcus aureus*	[132]
*Lavandula pubescens*	Shoots	2.5 M ZnCl_2_	10.76–20.42 nm	Rod-shaped	30 min	60 °C	*Pseudomonas aeruginosa* (ATCC 27853) and *Staphylococcus aureus* (ATCC 29213)	[133]
*Parthenium hysterophorus*	Leaves	1 mM zinc nitrate	10 nm	Spherical	8 h	90 °C	*Staphylococcus aureus*, *Streptococcus pneumoniae*, *Escherichia coli*, and *Klebsiella pneumoniae*	[134]
*Laurus nobilis*	Leaves	0.1 mol/L zinc acetate	29.983 nm	Spherical	1 h	Room temperature	*Staphylococcus aureus*, *S. epidermidis*, *Escherichia coli*, and *Klebsiella* spp.	[135]
*Lepidium sativum*	Seed	zinc nitrate	24.2 nm	Hexagonal	75 min	-	*Escherichia coli* and *Staphylococcus aureus*	[136]
*Averrhoa bilimbi*	Fruit	Zn(NO_3_)_2_·4H_2_O	35.4 to 59.5 nm	Round shape	5 h	70 °C	*Escherichia coli*	[137]
*Aloe vera*	leaves	10 mM ZnSO_4_·7H_2_O	50 to 220 nm	Hexagonal	24 h	Room temperature	*Staphylococcus epidermidis*, *Staphylococcus aureus*, *K. pneumoniae*, and *Escherichia coli*	[138]
*Ocimum lamifolium*	Leaves	0.06 M zinc acetate	22.8 nm	Spherical	2 h	pH 12 30 °C	*E. coli*, *S. aureus*, *P. aeruginosa*, and *S. pyogen*	[139]
*Ocimum tenuiflorum and Ocimum sanctum*	Leaves	30 mM zinc nitrate	-	-	10 min	Room temperature	*Streptococcus mutans*, *Enterococcus faecalis*, *Staphylococcus aureus*, and *Lactobacillus*	[140]
*Orange peel*	Peel	2 g of zinc nitrate with 42.5 mL of Zn(NO_3_)_2_·6H_2_O	10–20 nm	Spherical	60 min	60 °C	*Escherichia coli* and *Staphylococcus aureus*	[141]
*Cinnamomum verum*	Fruit	zinc acetate dehydrate (0.1 M)	56–71 nm	Spherical	60 min	50 °C	*E. coli* O157:H7 (02–0628) and *L. monocytogenes* (ATCC 7644)	[52]
*Myristica fragrans*	Fruit	6.0 g in 100 mL of (Zn(NO_3_)_2_·2H_2_O)	41.23 nm	Spherical or elliptical	2 h	60 °C	*K. pneumoniae*, *E. coli*, *P. aeruginosa*, and *S. aureus*	[94]
*Pistacia lentiscus* L.	Leaf	0.1 mol/L zinc acetate dihydrate (Zn(CH_3_COO)_2_ and 2H_2_O)	33.90 nm	Dried cotton shape	30 min	pH 12 78 °C	*Staphylococcus aureus* (ATCC 6538), *Bacillus* *cereus* (ATCC 10876), *Escherichia coli* (ATCC 8739), and *Pseudomonas aeuroginosa*	[51]
*Cnidoscolus aconitifolius*	Leaves	0.5 M Zn(CH_3_COO)_2_	100 nm	Spherical	9 h	50 °C	*Escherichia coli* ATCC 35218 *and* ATCC 25922, *Pseudomonas aeruginosa* ATCC 27853, *Klebsiella pneumonia* ATCC 700603 and *Chromobacterium violaceum* ATCC 12472, *Staphylococcus aureus* ATCC 43300 and ATCC 29213, *Enterococcus faecalis* ATCC 51299, and *Bacillus cereus* ATCC 29212	[142]
*Avicennia marina*	Leaves	10 mM ZnS	-	-	24 h	Room temperature	*Klebsiella* sp., *Staphylococcus aureus*, and *Streptococcus mutans*	[143]
*Lawsonia inermis*	Leaves	Zn(NO_3_)_2_	100 nm	Hexagonal	2 h	Room temperature	*Escherichia coli*, *Pseudomonas aeruginosa*, *Staphylococcus aureus*, and *Bacillus subtilis*	[144]
*Euphorbia petiolata*	Leaves	1 M zinc nitrate	-	Spongy shape	2 h	80 °C	*Escherichia coli*	[145]
*Ailanthus altissima*	Fruit	10 g in 200 mL of [(ZnNO_3_)_2_·6H_2_O]	5 to 18 nm	Spherical	Not specified	27 °C	*Escherichia coli* and *Staphylococcus aureus*	[146]
*Matricaria chamomilla* L.	Flower	1 M zinc oxide	48.2 nm	Cubic structures	4 h	Room temp	*Xanthomonas oryzae pv. Oryzae*	[147]
*Lippia adoensis (Koseret)*	Leaves	0.45 M zinc acetate dihydrate	19.78 nm	Spherical	2 h	Room temp	*Staphylococcus aureus* and *Enterococcus faecalis* *and Gram-negative* (*Escherichia coli* and *Klebsiella pneumonia*)	[148]
*Malus pumila*	Fruit	0.2 M zinc acetate dihydrate	12 nm	Spherical/grain rice-like (ellipsoidal)/cylindrical, dumbbell and needle-like shape	2 h	pH 6 70 °C	*E. coli*, *K. pneumoniae* and *P. aeruginosa*	[149]
*Catharanthus roseus* L.	Flower	0.1 g in 10 mL of ZnCl2	-	Spherical	3	Room temp	*Staphylococcus aureus* B23 and *Pseudomonas aeruginosa* 424	[150]
*Berberis aristata*	Leaves	0.1 M zinc acetate dihydrate		Needle like	Not specified	70 °C	*Escherichia coli*, *Staphylococcus aureus*, *Klebsiella pneumoniae*, *Bacillus subtilis*, *Bacillus cereus*, and *Serratia marcescens*	[151]
*Bauhinia tomentosa*	Leaves	2 mM ZnSO_4_	22–94 nm	Hexagonal	4 days	-	*Escherichia coli* and *Pseudomonas aeruginosa*	[152]
*Trifolium pratense*	Flower	0.5 M ZnO	60–70 nm	-	24 h	90 °C	S. aureus ATCC 4163, E. coli ATCC 25922, and *P. aeruginosa* ATCC 6749	[153]
*Cassia alata*	Leaves	0.01 M Zinc acetate	60–80 nm	Spherical	3 h	80 °C	Escherichia coli	[154]
*Melia azedarach*	Leaves	0.006 M zinc nitrate hexahydrate (Zn(NO_3_)·6H_2_O)	33–96 nm	Hexagonal and spherical	24 h	50 °C	*Escherichia coli* (ATCC 25922), *Staphylococcus aureus* (ATCC 25923), *Pseudomonas aeruginosa* (ATCC 27853), *Sphingobacterium thalpophilum* (NCTC 11429), *Bacillus subtilis* (ATCC 6051), and *Klebsella pneumonia* (ATCC 13883)	[155]
*Salvadora persica* L.	Root	0.1 M zinc acetate dihydrate (Zn(CH_3_COO)_2_·2H_2_O)	165–287 nm	Spherical	4.5 h	pH 8 70 °C	*Staphylococcus aureus* NRRL B-767, *Acinetobacter baumannii* 2.3, *Escherichia coli* ATCC 25922, *Enterococcus faecalis* ATCC 29212, *Klebsiella pneumoniae* NRRL B-4420, *Proteus vulgaris* NRRL B-123, and *Streptococcus mutans* (wild type)	[156]
*Saussurea lappa*	Rhizome, root	1 M hexahydrate zinc nitrate	123.5 nm	Hexagonal	2 h	70–80 °C	*Streptococcus aureus*, *Bacillus subtilis*, *Sphingobacterium thalpophilum*, *Staphylococcus aureus*, *E. coli*, *Pseudomonas aeruginosa*, *Sphingobacterium* sp., *Acinetobacter* sp., and *Ochrobactrum* sp.	[157]
*Pongamia pinnata*	Leaves	0.1 M zinc nitrate hexahydrate	10 to 120 nm	Spherical	24 h	Room temp	*S. aureus* and *E. coli*	[158]
*Murraya koenigii*	Leaves	1 M zinc acetate	15 nm	Spherical	2 h	pH 12 35–40 °C	*Staphylococcus aureus*, *Bacillus subtilis*, and *Salmonella typhii*; *Escherichia coli*, and *Klebsiella pneumoniae*	[159]
*Peltophorum pterocarpum*	Flower	zinc nitrate	69.45 nm	Spherical and irregular shaped	-	80 °C	(*Bacillus cereus* (BC) ATCC 11778, *Bacillus subtilis* (BS) ATCC 6633, *Staphylococcus aureus* (SA) ATCC 29737, *Corynebacterium rubrum* (CR) ATCC 14898), (*Escherichia coli* (EC) NCIM2931, *Pseudomonas aeruginosa* (PA) ATCC 9027, *Klebsiella pneumoniae* (KP) NCIM2719, and *Salmonella typhimurium* (ST) ATCC 23564)	[160]
*Cinnamomum verum*	Bark	zinc nitrate hexahydrate	~45 nm	Spherical	-	45–60 °C	*Staphylococcus aureus* (MTCC 7443) and *Escherichia coli* (MTCC 7410)	[161]
*Tecoma castanifolia*	Leaves	1 mM zinc sulphate	70–75 nm	Spherical	4 days	Room temp	*Bacillus subtilis*, *Staphylococcus aureus*, *Escherichia coli*, and *Pseudomonas aeruginosa*	[162]
*Garcinia cambogia*	Leaves	0.01 M Zn (NO_3_)_2_·6H_2_O	131.5 nm	Rod-like/hexagonal	12 h	60 °C	*Escherichia coli* (*E. coli*) ATCC 10536 and *Staphylococcus aureus* (*S. aureus*) ATCC 6538	[163]
*Camellia sinensis*	Leaves	1.5 g of zinc acetate dihydrate in 20 mL	10–20 nm	Spherical rods/needle and particle-like	1 h	Room temp	*E. coli* and *S. aureus*	[164]
*Brassica oleracea var. botrytis*	Leaves	5 g of Zn(NO_3_)_2_·6H_2_O in 50 ml	52 nm	Flower like	24 h	70 °C	*K. pneumonia* and *E. coli*	[165]
*Scoparia Dulcis*	Leaves	2 g of Zn(NO_3_)_2_·6H_2_O in 60 ml	~20 nm	Pebble-like	-	60 °C	*Staphylococcus aureus*-902 and *E. coli*-443	[166]
*Piper guineense*	seeds	0.25 g of Zn(CH_3_COO)_2_·2H_2_O was dissolved in 25 mL	7.39 nm	Hexagonal/spherical	2 h	60 °C	*E. coli* (ATCC 25922)	[167]
*Lupinus albus* and *Lupinus pilosus*	Leaves	40 mM zinc nitrate	19.70 nm and 28.13 nm	Rod-like	30 min	90 °C	*E. coli*, *P. aeruginosa* and *S. aureus*	[168]
*Ceratonia* *siliqua* L.	Pods	0.1 M zinc acetate dehydrate	-	Spherical/hexagonal	2 h	80 °C	*Staphylococcus aureus* ATCC 25923, *Micrococcus luteus* NCIMB8166, *Salmonella enterica*, *Typhimurium* ATCC 1408, and *Escherichia coli* ATCC 35218	[169]
*Basella alba*	Leaves	0.1 M zinc acetate	28.64 nm	Spherical	24 h	60 °C	*Pseudomonas aeruginosa*, *Escherichia coli*, *Enterobacter aerogenes*, *Staphylococcus aureus*, and *Proteus vulgaris*	[170]
*Bergenia* *ciliata*	Rhizome	1 M zinc acetate dihydrate	30 nm	Flower shape	2 h	pH 12 60 °C	*Yersenia enterocolitica*, *Pseudomonas aeruginosa*, *Salmonella typhi*, *Escherichia coli*, *Staphylococcus aureus*, and *Bacillus* *subtilis*	[171]
